# Ethanol Extract of *Illicium henryi* Attenuates LPS-Induced Acute Kidney Injury in Mice via Regulating Inflammation and Oxidative Stress

**DOI:** 10.3390/nu11061412

**Published:** 2019-06-23

**Authors:** Md Sodrul Islam, Lingyan Miao, Hui Yu, Ziyi Han, Hongxiang Sun

**Affiliations:** 1 Key Laboratory of Animal Virology of Ministry of Agriculture, College of Animal Sciences, Zhejiang University, Hangzhou 310058, China; msislam@bsmrau.edu.bd (M.S.I.); 645355598@zju.edu.cn (L.M.); 17605032171@sina.cn (H.Y.); 15222671162@163.com (Z.H.); 2 Departments of Physiology and Pharmacology, Bangabandhu Sheikh Mujibur Rahman Agricultural University, Gazipur 1706, Bangladesh

**Keywords:** *Illicium henryi*, lipopolysaccharide, acute kidney injury, TLR4, NF-κB, Nrf2

## Abstract

The root bark of *Illicium henryi* has been used in traditional Chinese medicine to treat various diseases. Its ethanol extract (EEIH) was found to contain a large number of phenols and possess in vitro antioxidant activities. The present study aimed to investigate its protective effect against lipopolysaccharide (LPS)-induced acute kidney injury (AKI) in mice. BALB/c mice were intraperitoneally pretreated with EEIH for five days, and then LPS injection was applied to induce AKI. Blood samples and kidney tissues were collected and used for histopathology, biochemical assay, enzyme-linked immunosorbent assay (ELISA), quantitative real-time polymerase chain reaction (qRT-PCR), and Western blot analyses. EEIH not only significantly dose-dependently attenuated histological damage and reduced renal myeloperoxidase (MPO) activity (from 9.77 ± 0.73 to 0.84 ± 0.30 U/g tissue) but also decreased serum creatinine (from 55.60 ± 2.70 to 27.20 ± 2.39 µmol/L) and blood urea nitrogen (BUN) (from 29.95 ± 1.96 to 16.12 ± 1.24 mmol/L) levels in LPS-treated mice. EEIH also markedly dose-dependently inhibited mRNA expression and production of TNF-α (from 140.40 ± 5.15 to 84.74 ± 5.65 pg/mg), IL-1β (from 135.54 ± 8.20 to 77.15 ± 5.34 pg/mg), IL-6 (from 168.74 ± 7.23 to 119.16 ± 9.35 pg/mg), and COX-2 in renal tissue of LPS-treated mice via downregulating mRNA and protein expressions of toll-like receptor 4 (TLR4) and phosphorylation of nuclear factor-κB (NF-κB) p65. Moreover, EEIH significantly dose-dependently reduced malondialdehyde (MDA) (from 5.43 ± 0.43 to 2.80 ± 0.25 nmol/mg prot) and NO (from 1.01 ± 0.05 to 0.24 ± 0.05 µmol/g prot) levels and increased superoxide dismutase (SOD) (from 22.32 ± 2.92 to 47.59 ± 3.79 U/mg prot) and glutathione (GSH) (from 6.57 ± 0.53 to 16.89 ± 0.68 µmol/g prot) levels in renal tissue induced by LPS through upregulating mRNA expression of nuclear factor erythroid 2 related factor 2 (Nrf2). Furthermore, EEIH inhibited LPS-induced intracellular reactive oxygen species (ROS) production from RAW264.7 cells in a concentration-dependent manner. These results suggest that EEIH has protective effects against AKI in mice through regulating inflammation and oxidative stress.

## 1. Introduction

Acute kidney injury (AKI) is a global public health hazard which harmfully affects patients’ health and contributes to an approximated 1.4 million deaths annually [1]. Clinically, AKI is defined as a sudden and reversible worsening of kidney function [2]. AKI is related to an enhanced risk of chronic kidney disease (CKD), end-stage renal disease (ESRD), and cardiovascular disease [3,4] and is recognized as a common and important problem in clinics that contributes to deregulating volume and electrolyte homeostasis [5]. It is generally related to sepsis-associated endotoxemia, which leads to about 50% of the mortality in intensive care units [6]. Sepsis is a severe clinical syndrome induced via a dysregulated reaction of the host to infection [7]. Sepsis is recognized to be a main pathogenesis of AKI and accounts for almost 26%–50% of all AKI in developed nations and 7%–10% of main renal-disorder-related AKI [8]. AKI induces an intolerably high mortality rate in patients with sepsis compared with those without sepsis [9]. Every year, more than 30 million people globally are affected by sepsis, according to a 2018 WHO report [10]. Lipopolysaccharide (LPS) is a common constituent of Gram-negative bacterial cell walls and infusion/injection of LPS has widely been applied as a model of experimental sepsis-associated AKI [11]. It is notable that LPS is one of the most crucial sources of sepsis and is implied in sepsis-related AKI pathogenesis, which can contribute to a “cytokine storm”, escalated oxidative stress, renal hypoperfusion, low blood pressure, and eventually a gradual decrease in kidney function [12]. Sepsis is caused by excessive secretion of proinflammatory mediators, reactive oxygen species (ROS), and reactive nitrogen species (RNS) by the host–pathogen interactions [11]. Inflammation and oxidative stress play a primary role in the pathogenesis of sepsis-associated AKI [13]. The production of ROS induced by LPS could affect the pathophysiology of sepsis via regulating the innate immune response and inducing pathologic injury to cells as well as organs [9]. The innate immune response induces the expression inflammatory genes such as proinflammatory cytokines, inducible nitric oxide synthase (iNOS), and cyclooxygenase 2 (COX-2) by activating toll-like receptor 4 (TLR4)-mediated nuclear factor-κB (NF-κB) [14,15]. The latter leads to ROS-mediated peroxidation of lipids, injury to nucleic acids, and oxidation of proteins, which finally results in cell death and tissue damage [9,16]. Nuclear factor erythroid 2-related factor 2 (Nrf2) is a master controller of the cellular redox status in sepsis and is a major defense mechanism against LPS-induced oxidative renal stress [9]. Thus, appropriate drugs that suppress inflammation and oxidative stress might be favorable in treating sepsis-associated AKI.

Many natural products have been used to prevent and treat kidney disease [17]. The root bark of *Illicium henryi* Diels (Illiciaceae), commonly known as Chinese anise tree or hardwood crab, has been used for alleviating pain and swelling, removing phlegm, promoting blood circulation, and dispelling pathogenic wind, cold and dampness in traditional Chinese medicine [18,19]. Modern pharmacological studies have shown that this drug possesses anti-inflammatory, antioxidant, anti-hepatitis B virus, and anti-HIV activities [20,21]. The root bark of *I. henryi* has been reported to contain flavonoids, prenylated C6–C3 compounds, sesquiterpene lactones, triterpenoids, lignans, and neolignans [18,22,23]. Among these, flavonoids such as quercetin and its glycosides are considered to be the major active constituents of this crude drug and are responsible for the antioxidant and anti-inflammatory activities [20,24]. In a previous study, the ethanol extract of *I. henryi* root bark (EEIH) was found to contain a large number of phenolic compounds and possess 2, 2-diphenyl-1-picrylhydrazil (DPPH) and 2,2′-azino-bis-(3-ethylbenzothiozoline-6-sulfonic acid) disodium salt (ABTS) radical scavenging activities and ferric-reducing antioxidant capacity in vitro (Table S1). Therefore, EEIH would be beneficial against acute kidney injury. The aim of this study was to evaluate the renoprotective effects of EEIH on LPS-induced kidney injury in mice and explore its molecular mechanisms from the perspective of antioxidant and anti-inflammatory abilities in vivo.

## 2. Materials and Methods

### 2.1. Chemical and Reagents

LPS (from *Escherichia coli* 055:B5) (#L2880) was purchased from Sigma-Aldrich Chemical Co., St. Louis, MO, USA; TNF-α (#EK0527), IL-1β (#EK0394), and IL-6 (#EK0411) ELISA kits were from Wuhan Boster Biological Technology Co. Ltd., Wuhan, Hubei, China; glutathione (GSH) (#A006-2), malondialdehyde (MDA) (#A003-1), myeloperoxidase (MPO) (#A044), nitric oxide (NO) (#A013-2), and superoxide dismutase (SOD) (#A001-3) assay kits were from Jiancheng Bioengineering Institute, Nanjing, Jiangsu, China; TRIzol reagent (#10296010) was from Invitrogen, Carlsbad, CA, USA; revert Aid™ M-MLV reverse transcriptase (#EP0441) was from Fermentas, Amherst, NY, USA; ribonuclease inhibitor and oligo(dT)_18_ (#B300816) were from Sangon Biotech (Shanghai) Co. Ltd., Shanghai, China; FastStart universal SYBR Green Master (ROX) (#4913850001) was from Roche Diagnostics, Indianapolis, IN, USA; BCA protein assay kit (#P0012S), RIPA lysis buffer (#P0013B), 10% sodium dodecyl sulfate polyacrylamide gel electrophoresis (SDS-PAGE) (#P0690), HRP-conjugated goat anti-rabbit and anti-mouse IgG (H+L) (#A0208 and #A0216), BeyoECL Star kit (#P0018AS), and ROS assay kit (#S0033) were purchased from Beyotime Biotechnology, Shanghai, China; phosphatase inhibitor cocktail (#B15001) and protease inhibitor cocktail (#B14001) were from Bimake, Houston, TX, USA; anti-rabbit NF-κB p65 (C-20) (#sc-8008) and TLR4 (#sc-293072) polyclonal antibodies were from Santa Cruz Biotechnology, Dallas, TX, USA; anti-mouse actin monoclonal antibody (#3700) and anti-rabbit phospho-NF-κB p65 (Ser536) polyclonal antibody (#3033) were from Cell Signaling Technology, Beverly, MA, USA; and dexamethasone (DEX) (#30049090) was provided by Hubei Tianyao Pharmaceutical Co., Ltd., Xiangyang, China.

### 2.2. Preparation and Characterization of EEIH

The dry root bark of *I. henryi* was purchased from Yinghuitang Pharmaceutical Co. Ltd., Bozhou, Anhui, China in May 2018 and authenticated by Prof. Yonghai Jiang from the Shaoxing Institute for Food and Drug Control, Zhejiang, China. A voucher specimen (no. 20180506) was deposited in the laboratory of natural medicine, College of Animal Sciences, Zhejiang University, Hangzhou, China. The powdered material (1 kg) was extracted with eightfold 75% ethanol twice under reflux for 2 h. After filtration and centrifugation (1700× *g*, 30 min), the combined solution was concentrated under reduced pressure in a rotavapor at 45 °C to evaporate the solvent and then lyophilized to afford ca. 91.68 g of EEIH (yield 9.17% *w*/*w*). The total phenolic and flavonoid contents in EEIH were measured to be 49.67% ± 0.32% and 41.44% ± 0.27% by the Folin–Ciocalteu method and a spectrophotometric method using gallic acid and rutin as the control standards, respectively. 

### 2.3. Cell Culture

RAW264.7 macrophages were purchased from a cell bank, Shanghai Branch of the Chinese Academy of Sciences, and maintained in a 5% CO_2_ atmosphere in DMEM medium supplemented with 10% FCS, 50 μM mercaptoethanol, 20 mM HEPES, 10 mM sodium pyruvate, 100 μg/mL streptomycin, and 100 U/mL penicillin.

### 2.4. Experimental Animals

Male BALB/c mice aged 5–6 weeks were purchased from Shanghai Experimental Animal Center of Chinese Academy of Sciences, Shanghai, China (certificate no. SCXK 2007-0005). Mice were acclimatized for 1 week prior to use. Rodent laboratory chow and tap water were provided ad libitum and maintained under controlled conditions with a temperature of 24 ± 1 °C, humidity of 50% ± 10%, and a 12/12 h light/dark cycle. All the procedures were in strict accordance with PR China legislation on the use and care of laboratory animals and the guidelines established by the Institute for Experimental Animals of Zhejiang University and were approved by the university committee for animal experiments (no. 14878).

### 2.5. Treatment of Mice

Male BALB/c mice were divided into six groups: normal control group (NC), model control group (MC), positive control group (DEX), and EEIH groups (1.25, 2.5, and 5.0 mg/kg). Each group consisted of five mice. The doses of EEIH were used based on our preliminary study. The mice were intraperitoneally injected with EEIH at the doses of 1.25, 2.5, and 5.0 mg/kg or with 1.8 mg/kg of DEX every day for 5 days. The normal control and model control groups received the same volume of saline. The dose volume was 0.2 mL/10 g body weight. One hour after the last administration, all mice except for the normal control group were intraperitoneally administered with LPS (8 mg/kg) [25], while the control mice merely received saline. After fasting for 12 h, all mice were weighed and anesthetized by 10% chloral hydrate. Blood was drawn from the eyes and the serum was prepared by centrifuging at 3500 rpm for 10 min at 4 °C. The mice were then sacrificed via cervical dislocation, and the renal tissues were collected, weighed, washed with saline (0.9% NaCl), and stored at −80 °C until further analysis. The renal index was measured utilizing the following formula: kidney weight/mouse body weight × 100 (g/100 g body weight).

### 2.6. Kidney Histopathology

The collected kidney tissues were immediately fixed with 4% paraformaldehyde. Then, the fixed renal tissues were dehydrated through a graded series of ethanol, hyalinized with xylene, embedded in paraffin, and sectioned at 5 μm thicknesses. A series of microsections was stained with haematoxylin and eosin (H&E) for histological assessment. Digital images were obtained using an Olympus CKX53 microscope at a fixed 100× magnification.

### 2.7. Biochemical Examinations of Serum Creatinine (Cre) and Blood Urea Nitrogen (BUN) Levels

The serum Cre and BUN levels were measured using a Roche Cobas C311 Chemistry Analyzer (Roche Diagnostics, Indianapolis, IN, USA). The values were expressed as µmol/L or mmol/L of sera.

### 2.8. Measurement of Renal MDA, MPO, NO, SOD, and GSH Levels or Activities

Renal tissue samples (ca. 70 mg) were mixed with 630 μL of PBS to prepare 10% homogenate by a tissue homogenizer (Shanghai, China). The homogenate supernatants were collected by centrifuging at 12,000 rpm for 10 min at 4 °C. The concentration of protein in the supernatants was measured using the BCA assay kit. The collected homogenate (for MPO) or supernatants (for other indices) were measured for renal MDA, MPO, NO, SOD, and GSH levels or activities by spectrophotometry using commercial diagnostic kits according to the manufacturer’s instructions. The protein concentrations in the supernatants were also detected with the BCA method using bovine serum albumin as a standard. The renal MDA, MPO, NO, SOD, and GSH levels were standardized to the protein in each sample. MPO level was expressed as U/gram tissue, MDA level was as nmol/mg of protein, GSH level was as µmol/gram of protein, SOD level was as U/mg of protein, and NO level was as µmol/gram of protein.

### 2.9. ELISA

The concentrations of TNF-α, IL-1β, and IL-6 in the above homogenate supernatants were detected using commercial ELISA kits as previously described [26]. The values were expressed as pg/mg of protein based on appropriate standard curve.

### 2.10. Quantitative Real-Time PCR (qRT-PCR)

The total RNA was isolated from homogenized kidney tissues using the Trizol reagent according to the manufacturer’s protocol, and reverse transcription was performed as previously described [27]. Then, amplification was carried out in a 20 μL reaction using FastStart Universal SYBR Green Master. Real-time PCR was performed using the Bio-Rad CFX96 real-time PCR system. Primers for qRT-PCR were synthesized by Sangon Biotech (Shanghai) Co., LTD, Shanghai, China and the sequences are listed in Table 1. The qPCR cycling was performed as follows: initial denaturation at 95 °C for 10 min, followed by 40 cycles of denaturation at 95 °C for 10 s, and then annealing at 60 °C for 1 min. GAPDH was used as an endogenous control. Primer amplification efficiency and specificity were verified for each set of primers. The expression levels of the tested genes relative to GAPDH were determined using the 2^ΔΔCt^ method and as fold induction [27].

### 2.11. Western Blotting

The harvested renal tissues were homogenized in RIPA lysis buffer supplemented with protease and phosphatase inhibitors (cocktail) and centrifuged at 12,000 rpm for 15 min at 4 °C. The protein contents were measured with the BCA protein assay kit. The denatured proteins were separated on 10% SDS-PAGE and then transferred to a polyvinylidene fluoride (PVDF) membrane (0.45 μM). After blocking the membrane with 5% skim milk in Tris-buffered saline containing 0.1% Tween-20 (TTBS) for 2 h at 37 °C, the blot was incubated with anti-mouse actin monoclonal antibody, anti-rabbit TLR4, NF-κB p65, or phospho-NF-κB p65 polyclonal antibody in TTBS containing 5% skim milk overnight at 4 °C. The membranes were washed with TTBS and then incubated with horseradish-peroxidase-conjugated goat anti-mouse or anti-rabbit IgG for 2 h. After washing the membrane with TTBS three times, the signal was visualized with BeyoECL Star kit using the LiCor C-DiGit Blot Scanner (LiCor, Lincoln, NE, USA).

### 2.12. MTT Assay

The effect of EEIH on the growth of RAW264.7 cells was determined by the MTT method. RAW264.7 cells were seeded at 2 × 10^4^ cell/well in a 96-well plate and incubated at 37 °C in a humidified atmosphere with 5% CO_2_. After 24 h, the various concentrations of EEIH were added into each well and these cells were incubated at 37 °C. After 20 h, the proliferation was detected using the MTT assay as previously described [26]. 

### 2.13. ROS Detection

The intracellular ROS levels were detected using the ROS assay kit according to the manufacturer’s protocol. Briefly, cells were plated at 2 × 10^5^ cell/well in 24-well plates and pretreated with different concentrations of EEIH (50, 100, and 200 μg/mL) for 24 h. After treatment with LPS (0.1 µg/mL) for 2 h, the cells were incubated with 20 μM 2′,7′-dichlorofluorescin diacetate (DCFH-DA) at 37 °C for 30 min in the dark and then washed with DMEM three times. The intensity of fluorescence was determined by flow cytometry with the BD FACSVerse system at 488 nm excitation and 525 nm emission wavelengths.

### 2.14. Statistical Analysis

The normality of the distribution of each variable was measured through means of the Kolmogorov–Smirnov test. The data were expressed as mean ± standard deviation (SD) and examined for their statistical significance of difference with ANOVA and Student’s *t*-test. The calculations and graphs were produced using Prism 7 software (GraphPad Software, USA). *P*-values of less than 0.05 were considered to be statistically significant.

## 3. Results

### 3.1. Effects of EEIH on Body Weight and Renal Index in LPS-Treated Mice

The effect of EEIH on body weight was firstly assessed. As shown in Table 2, there were no significant differences among the various groups (*p* > 0.05). However, the kidney index of mice in the MC group was significantly higher than that in the NC group (*p* < 0.05). Pretreatment with DEX and EEIH at 5 mg/kg remarkably decreased the renal index of LPS-treated mice (*p* < 0.05). However, no differences were found in the renal index among the EEIH groups (1.25 and 2.5 mg/kg) and MC group (*p* > 0.05). The results suggest that EEIH could dose-dependently suppress the enlargement of the renal index in mice with LPS-induced kidney injury.

### 3.2. EEIH Attenuated Histological Damage and Reduced MPO Activities in Renal Tissue of LPS-Treated Mice

**Figure 1:**
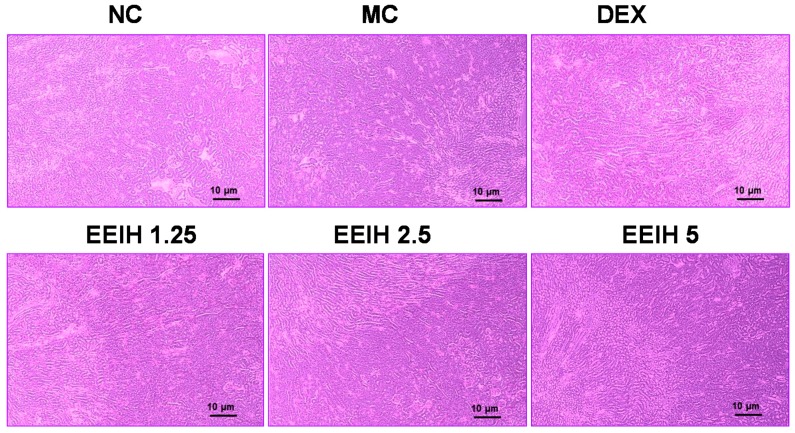
Effects of EEIH on histopathological changes of renal tissue in LPS-treated mice. The kidney sections were stained using hematoxylin and eosin. The light photomicrographs shown are representative of kidney sections from five mice per group. NC: normal control; MC: model control; DEX: dexamethasone (positive control).

Histological examination and MPO detection were carried out to evaluate the degree of injury of kidney tissue in LPS-treated mice. As shown in Figure 1, the renal tissues in normal control mice displayed normal renal cellular architecture, a visible nucleus, as well as no apparent inflammation. Conversely, a remarkable kidney pathological anomaly was found in the MC group, characterized by edema of renal tubular epithelial cells, tubular dilatation and distortion, infiltration of inflammatory cells, as well as damage of glomerular structure, indicating that the LPS-induced AKI mouse model in this study was successful. However, pretreatment with EEIH (1.25, 2.5, and 5 mg/kg) or DEX notably improved the anomalous histopathological alterations in mice induced by LPS. Neutrophils are the first line of innate immune defense against infection. In order to further assess the effects of EEIH on LPS-induced inflammation, MPO activity in kidney tissue was examined using commercial diagnostic kits. LPS significantly promoted the activity of the neutrophil sequestration indicator MPO in renal tissue of model mice compared to the normal control group (*p* < 0.001). Pretreatment with EEIH at three doses and DEX markedly decreased the renal MPO activity in LPS-treated mice (*p* < 0.001; Table 3). These findings suggest that EEIH has the ability to protect against LPS-induced AKI in mice.

### 3.3. EEIH Reduced the Serum Cre and BUN Levels in LPS-Induced AKI Mice

Serum Cre and BUN levels, as crucial and primary indicators of kidney damage severity, were used to evaluate kidney function [2]. To evaluate the effects of EEIH on renal function, the level of serum Cre and BUN were examined and the results are shown in Table 3. Significant increases in the serum Cre and BUN levels were found in the LPS-induced AKI mice compared with the normal control group (*p* < 0.001). However, the elevated levels of serum Cre and BUN in LPS-treated mice were notably inhibited by EEIH at three dose (1.25, 2.5, and 5 mg/kg) and positive drug DEX (*p* < 0.001). The results indicate that EEIH could dose-dependently suppress the enhancement of serum Cre and BUN levels in mice with LPS-induced kidney injury.

### 3.4. EEIH Exerted Anti-Inflammatory Activity in Renal Tissue of LPS-Induced AKI Mice via TLR4/NF-κB Pathway

During the initial stage of infection, LPS causes a “cytokine storm” [28]. Upregulation of proinflammatory cytokines might be linked to the level of tissue injury and correlated inflammatory responses. The pathogenesis of LPS-induced AKI is related to the elevation of proinflammatory cytokines such as TNF-α, IL-1β, and IL-6 [14]. Therefore, the levels of TNF-α, IL-1β, and IL-6 in kidney tissue were measured, and the results are shown in Table 4. LPS led to a significant increase in the level of TNF-α (*p* < 0.001), IL-1β (*p* < 0.001), and IL-6 (*p* < 0.001) in kidney tissue compared with the normal control group. However, the levels of TNF-α (*p* < 0.001), IL-1β (*p* < 0.01 or 0.001), and IL-6 (*p* < 0.01–0.001) in kidney tissue of LPS-treated mice were significantly reduced by pretreatment with EEIH in a dose-dependent manner and DEX. These results indicate that EEIH could suppress the production of inflammatory cytokines from the kidney tissues of LPS-induced AKI mice. 

Furthermore, the mRNA expression levels of these proinflammatory factors were also determined by qRT-PCR. As shown in Table 5, LPS remarkably upregulated the mRNA expression levels of TNF-α, 1L-1β, IL-6, and COX-2 compared with the NC group (*p* < 0.001). However, pretreatment with EEIH at three doses and DEX significantly downregulated the mRNA expression levels of TNF-α, IL-1β, IL-6, and COX-2 in renal tissue of LPS-treated mice (*p* < 0.001). These findings confirmed the anti-inflammatory mechanism of the protective activity of EEIH against LPS-induced AKI in mice. 

**Figure 2:**
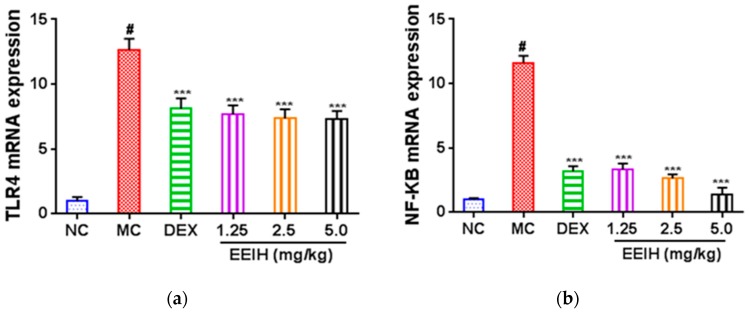

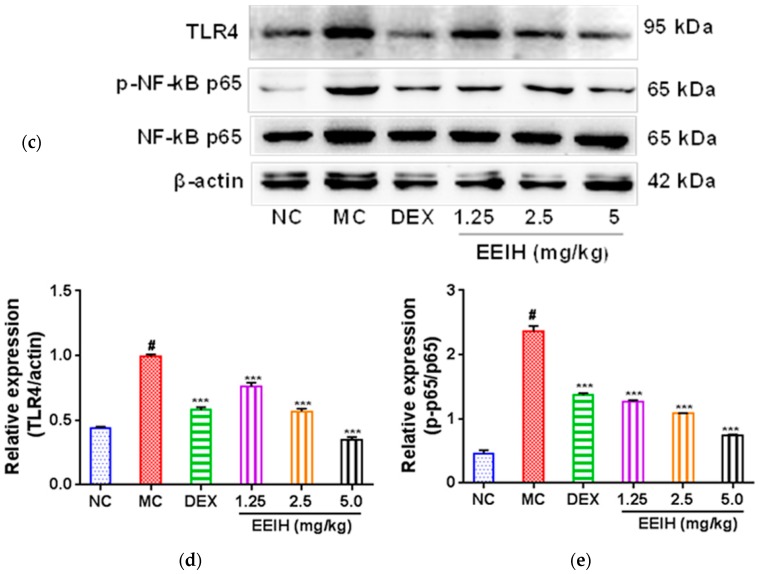
Effects of EEIH on the mRNA (**a**,**b**) and protein (**c**–**e**) expression levels of toll-like receptor 4 (TLR4) and nuclear factor-κB (NF-κB) in renal tissue of LPS-treated mice. The figure shown is representative of three independent experiments. The values are presented as the means ± SD (*n* = 5). Significant differences compared to the normal control group (NC) are designated as ^#^ *p* < 0.001; those compared to the model control group (MC) as *** *p* < 0.001. DEX: dexamethasone (positive control).

TLR4-mediated NF-κB signaling plays a critical role in the LPS-induced expression of inflammatory factors [14,15]. To explore the inhibitory mechanism of EEIH against inflammatory responses, the mRNA expression levels of TLR4 and NF-κB in renal tissue were first determined by qRT-PCR, and the results are shown in Figure 2a,b. The mRNA expression levels of TLR4 and NF-κB p65 were drastically upregulated in the renal tissue of LPS-treated mice compared with the normal control group (*p* < 0.001). However, EEIH and DEX notably reduced the upregulated expression levels of TLR4 and NF-κB p65 mRNAs in renal tissue of LPS-treated mice (*p* < 0.001). Furthermore, the protein expression of TLR4 and the phosphorylation of NF-κB p65 in the renal tissue of LPS-treated mice were also detected using Western blot. LPS significantly induced the protein expression of TLR4 and the phosphorylation of NF-κB p65 (Figure 2c–e). Pretreatment with EEIH at three doses and DEX significantly inhibited the protein levels of TLR4 and the phosphorylation of NF-κB p65 in the renal tissue of LPS-induced AKI mice. These results suggest that EEIH exerts anti-inflammatory activity via the LPS-activated TLR4/NF-κB pathway. 

### 3.5. EEIH Relieved Kidney Oxidative and Nitrosative Stress in Mice Induced by LPS through Upregulating Nrf2 mRNA Expression

LPS-induced renal damage interrupts the intracellular redox balance and induces oxidative stress [11]. The reduction of endogenous antioxidants such as SOD and GSH and the elevation of MDA have been implied in the pathological process of AKI [11]. A previous report showed that LPS-induced AKI could be mitigated via oxidative stress suppression [12]. In this study, LPS significantly descreased the SOD activity and GSH level and increased the MDA level in the renal tissue of mice compared with the normal control mice (*p* < 0.001, Table 6). However, pretreatment with EEIH and DEX drastically enhanced SOD activity and GSH level as well as reduced the MDA level in the renal tissue of LPS-treated mice (*p* < 0.001). Similarly, the NO levels in the renal tissue of LPS-treated mice were markably reduced by EEIH at three doses and DEX, almost close to that of normal control mice (Table 6). 

The mRNA expression level of iNOS in renal tissue was further measured, and the results are shown in Table 7. LPS significantly upregulated the mRNA expression levels of iNOS in renal tissue of mice comared with the normal control mice (*p* < 0.001). The pretreatmet of EEIH resulted in marked inhibition of the mRNA expression of iNOS in renal tissue of the LPS-treated mice (*p* < 0.001). These results suggest that EEIH intervention notably relieves oxidative and nitrosative stress. Nrf2 is a master controller of the cellular redox status in sepsis and is the major defense mechanism against LPS-induced oxidative renal stress [9]. To investigate the role of oxidative stress in EEIH-mediated benefits, the expression level of Nrf2 mRNA in renal tissue was further measured using qRT-PCR. As shown in Table 7, a significant increase in the mRNA expression level of Nrf2 was found in renal tissue of LPS-treated mice compared with the normal control mice (*p* < 0.001). Further, the pretreatment of EEIH and DEX further accelerated the mRNA expression of Nrf2 in renal tissue of LPS-treated mice. The results suggest that EEIH relieves the renal oxidative and nitrosative stress burden behind the observed nephroprotective activity.

### 3.6. EEIH Inhibited ROS Production in RAW264.7 Cells

**Figure 3:**
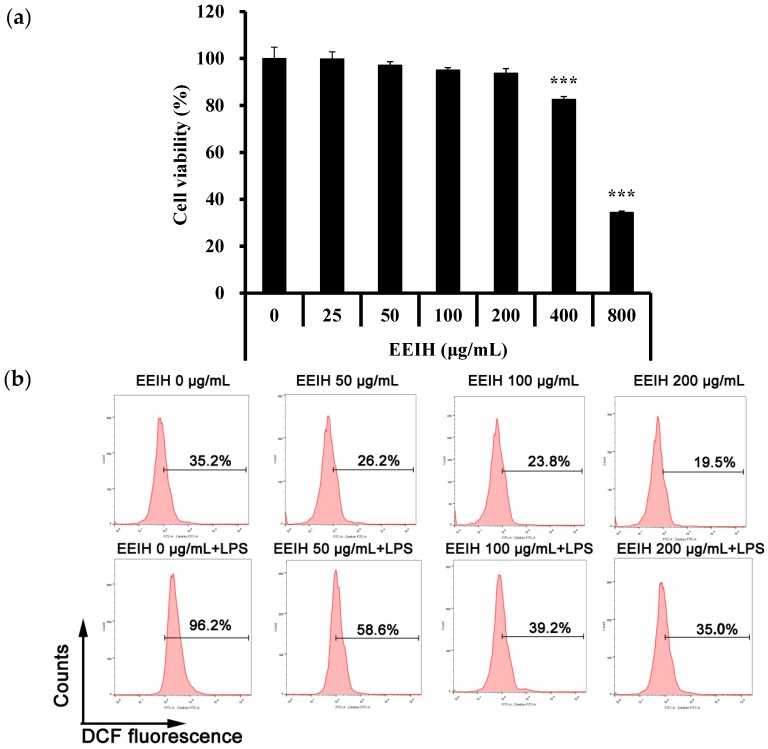
Effect of EEIH on viability (**a**) and reactive oxygen species (ROS) production (**b**) of RAW264.7 cells. The values are presented as mean ± SD (*n* = 3). Significant differences with control cells were designated as *** *p* < 0.001. The figure shown is representative of three independent experiments.

To examine the cell viability of EEIH, RAW264.7 cells were cultured with different concentrations of EEIH (25–800 µg/mL) for 24 h. EEIH at a concentration > 200 µg/mL notably decreased the viability of RAW264.7 cells (Figure 3a). Therefore, the concentrations of 50, 100, and 200 μg/mL were selected for evaluating the effects of EEIH on intracellular ROS generation in RAW264.7 cells. As shown in Figure 3b, LPS remarkably induced the production of ROS from RAW264.7 cells. However, the pretreatment with EEIH significantly concentration-dependently decreased the intracellular ROS levels in RAW264.7 cells treated with and without LPS (Figure 3b), confirming that EEIH attenuated oxidative stress in RAW264.7 cells induced by LPS.

## 4. Discussion

LPS has often been used to establish acute renal injury mouse models, which exhibit similar pathological changes to those found in human infectious sepsis [12,29]. LPS-induced AKI is characterized by oxidative stress, inflammation, as well as enhanced serum creatinine and urea levels. In the present study, the nephroprotective effects of EEIH were investigated using an LPS-induced AKI mouse model. EEIH significantly decreased the levels of serum Cre, BUN, inflammatory factors (TNF-α, IL-6, IL-1β, and COX-2), MDA, and NO and enhanced the kidney antioxidant activities of SOD and GSH in LPS-treated mice; also, it inhibited ROS generation in LPS-stimulated RAW264.7 cells. Further, EEIH effectively improved the histological changes and MPO activity in renal tissues of LPS-treated mice. The qPCR and Western blot analysis demonstrated that EEIH ameliorated LPS-induced acute renal damage through increasing antioxidant defense and inhibiting inflammatory events by Nrf2 and TLR4-NF-kB signaling pathways. The Kolmogorov–Smirnov test was performed using R to check the normality of the datasets. It was observed that *p*-values were > 0.05 for all datasets. Therefore, parametric tests were used for the datasets.

The concentrations of serum Cre and BUN are important indicators of kidney injury [30,31]. In this study, the significant enhancements in the serum Cre and BUN levels in LPS-treated mice indicated the damaged structural integrity of nephrocytes and renal dysfunction [29]. Pretreatment with EEIH was found to decrease serum Cre and BUN levels, probably via keeping the integrity of the cell membrane, which agreed with a previous study [32]. This result was also verified by histopathological observation, which was used to assess the damage of the kidney structure [33]. The pretreatment with EEIH revealed a significant effect in preserving the renal cell architecture and decreasing inflammatory cell infiltration. Neutrophil infiltration is a vital step in the development of organ dysfunction. MPO is an indicator of neutrophil infiltration as well as severity of inflammation during sepsis [34]. The aggregation of neutrophil in renal tissue revealed the degree of inflammation. Pretreatment with EEIH resulted in a remarkable decline in the MPO level in renal tissue of LPS-induced AKI mice, which was in agreement with a previous study [14]. These results suggest that EEIH leads to the optimal protective effect on renal tissue via restricting the degree of inflammation. In addition, EEIH could dose-dependently decrease the renal index of LPS-induced AKI mice. This phenomenon might be attributed to the improvement of renal dysfunction by EEIH treatment.

Proinflammatory cytokine or mediators are known to play a significant role in the pathogenesis of sepsis-associated AKI. The massive production of these cytokines can induce kidney injury [13]. TNF-α is a crucial mediator which can induce renal damage through triggering TNF receptors [35]. IL-1β plays a major role in local acute inflammation, which is regarded as a panic cytokine of sepsis [36]. IL-6 plays a vital role in acute renal damage, which is regarded to be a predictor of AKI in patients with serious sepsis [37]. In the present study, LPS markedly induced the secretion of proinflammatory cytokines TNF-α, IL-1β, and IL-6 in renal tissues. However, EEIH notably diminished the production and mRNA expression levels of TNF-α, IL-1β, and IL-6 in renal tissue of LPS-induced AKI mice. COX-2 plays a crucial role in the pathophysiology of inflammation and serves a favorable function under the state of oxidative stress in the renal tissue, and ROS might induce tissue injury through modulating COX-2 to cause inflammatory responses [38]. Similarly, pretreatment with EEIH also downregulated the mRNA expression level of COX-2 in renal tissue of LPS-treated mice. These findings suggest that EEIH might alleviate renal damage through its anti-inflammatory activities. 

TLR4 is the primary receptor of LPS and has a central role in the innate immune process [39]. Stimulation of TLR4 with LPS activates NF-κB, contributing to the production and secretion of inflammatory cytokines with consequent renal injury [13,14]. It was reported that TLR4 in the kidney is a crucial donor of innate stimulation and inflammation which contributes to renal injury [15,40]. An increased level of TLR4 was found in septic patients [41]. Recent evidence has shown that TLR4 deficiency increases resistance in sepsis-induced immune dysfunction [42]. TLR4 was recently reported to be massively expressed in LPS-induced AKI [43]. To further understand the molecular mechanism through which EEIH exerts its anti-inflammatory activity, the mRNA and protein expression levels of TLR4 were assessed using qRT-PCR and Western blot, respectively. The results showed that pretreatment with EEIH significantly dose-dependently downregulated the mRNA and protein expression levels of TLR4 in renal tissue of LPS-induced AKI mice, which was consistent with earlier studies [14,44]. The expression level of TLR4 was proved to be correlated with the levels of inflammatory cytokines in renal tissue of LPS-induced AKI mice [45,46]. Activation of TLR4 in mice by LPS could activate an NF-κB cascade in the kidney, which leads to propagation and expression of inflammatory cytokines, resulting in further renal damage [13]. NF-κB is a crucial transcriptional factor that is a central link between the pathogenesis of inflammation and the renal damage induced by LPS [47]. Moreover, it was recently reported that NF-κB could modulate various gene expression levels which are associated with the pathophysiology of sepsis [48]. Activation of NF-κB is linked to LPS-induced mortality in animals. Suppression of NF-κB activation markedly decreased the mortality of LPS-treated animal models [49] and patients [50] and elevated the tolerance to endotoxins [51]. Furthermore, suppression of TLR4 and NF-κB-induced inflammatory reaction has been corroborated to have renoprotective effects against LPS-induced AKI [14,52]. In this study, pretreatment with EEIH significantly downregulated the mRNA expression level and phosphorylation of NF-κB p65 in the renal tissue of LPS-treated mice. These results suggest that EEIH has the ability to mitigate renal inflammation via regulating TLR4 expression and NF-κB activation as well as consequently suppressing the production of inflammatory cytokines, which is in agreement with previous studies [14,52].

Oxidative stress is one of the major hallmarks of AKI progression and is characterized by an accumulation of elevated ROS as well as impaired antioxidant capability [53]. There is a noticeable positive link between the level of oxidative stress and the severity of kidney injury [16]. LPS was reported to induce ROS production and inhibit the antioxidant defense process, which contributes to oxidative renal tissue damage and tubular structures, culminating in AKI [11]. Overall, the production of free radicals is eliminated through intracellular antioxidant enzymes such as GSH and SOD, which are relatable antioxidants in kidney cells, thus controlling the production and elimination of free radicals to be balanced [54]. SOD, as the essential scavenger of ROS, can catalyze the extremely reactive superoxide anion O_2_^−^ into O_2_ and hydrogen peroxide (H_2_O_2_) [55]. GSH can protect cells from oxidative injuries through efficiently eradicating lipids as well as other organic peroxides [56]. A reduced SOD level in renal tissue indicates that the kidney was not protected from ROS (O_2_^−^)-induced oxidative renal damage in sepsis [57]. In this study, EEIH pretreatment significantly restored the reduced SOD and GSH activities in renal tissue induced by LPS, suggesting the free-radical-scavenging and antioxidant activity of EEIH. This process was further confirmed by MDA, a final product of lipid peroxidation, which is extensively considered to be an indicator of ROS for measuring the level of oxidative stress in the kidney [11]. LPS enhanced ROS levels and accelerated lipid-peroxidation-mediated cytotoxic MDA formation, leading to oxidative renal damage [9,11]. Pretreatment with EEIH remarkably decreased the MDA levels in renal tissues of LPS-induced AKI mice, further confirming that EEIH has the capacity to relieve renal oxidative stress. These in vivo results correlated well with the in vitro antioxidant experiment, which demonstrated a significant ferric-reducing ability, suggesting that EEIH has potent antioxidant activity. EEIH also showed a potential antioxidant ability to scavenge DPPH radicals, while the ABTS radical scavenging activity was more sensitive compared with DPPH radicals or ferric ions (Table S1). In addition, EEIH also concentration-dependently inhibited LPS-induced intracellular ROS production in RAW264.7 cells. This was attributed to the presence of a significant amount of phenolic compounds and flavonoids in EEIH [58,59].

The expression of iNOS was induced in vascular endothelial cells to accelerate the production of high NO [60]. The massive accumulation of NO contributes to the formation of extratoxic peroxynitrite after the response with superoxide anions (O_2_^−^) to cause injury to cell membranes, proteins, and DNA, which ultimately results in nitrosative stress [61]. LPS administration evoked a substantial elevation in renal tissue nitric oxide, prompting nitrosative stress and subsequent kidney toxicity [11]. EEIH significantly inhibited the production of NO and the mRNA expression of iNOS in renal tissue of LPS-induced AKI mice, which was consistent with a recent study [11]. These findings suggest that EEIH possesses protective activity against renal nitrosative stresses and consequently ameliorates kidney dysfunction.

Nrf2 is a redox-sensitive transcription factor that primarily expresses in metabolic and detoxifying kidney tissues and defends cells from oxidative stress [62]. Under oxidative stimuli, Nrf2 is detached from the cytoplasm, transfers to the nucleus, and binds to the antioxidant response element (ARE) site to raise the endogenous phase II detoxifying and antioxidant enzyme expression, such as SOD, CAT, and GSH-Px [63]. The activation of Nrf2 in the kidney implies the removal of toxins and ROS that are probably essential in kidney toxicity [64]. In the present study, LPS significantly upregulated the mRNA expression level of Nrf2 in renal tissue, which was in agreement with earlier studies [9,44]. This might be due to the activation of kidney self-defense against oxidative stress induced by LPS. Pretreatment with EEIH, however, further reinforced the expression of Nrf2 mRNA upregulation, indicating that EEIH might be at least partially attributed to the amelioration of renal oxidative stress damage by an Nrf2-mediated mechanism. Moreover, the anti-inflammatory activity of EEIH might be linked to its capacity to elevate Nrf2. In this context, the absence of the Nrf2 gene in mice prompts oxidative stress, nitrosative stress, inflammation, as well as kidney damage in diabetic mice [65]. Moreover, Nrf2 could inhibit NF-κB, NO, and proinflammatory cytokine levels in LPS-induced AKI [9]. Here, EEIH might exert antioxidative activities through upregulating the mRNA expression of Nrf2.

## 5. Conclusions

**Figure 4:**
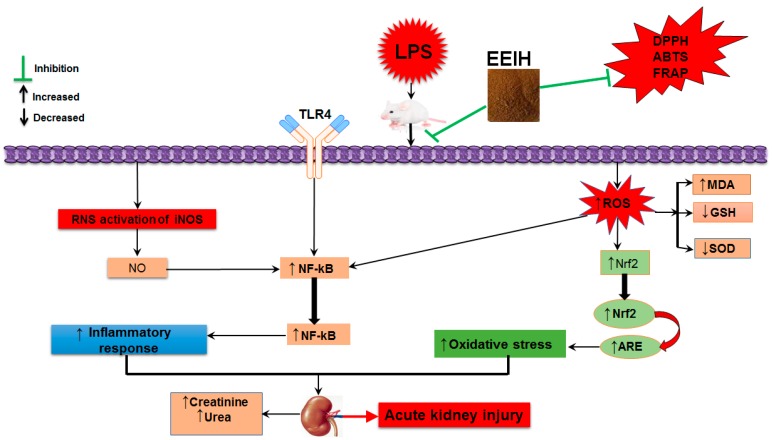
A proposed schematic diagram illustrating the protective effect of EEIH on LPS-induced acute kidney injury. EEIH, ethanol extract of *Illicium henryi*; LPS, lipopolysaccharide; DPPH, (2, 2-diphenyl-1-picrylhydrazil); FRAP, ferric-reducing antioxidant power; ABTS, 2,2′-azino-bis-(3-ethylbenzothiozoline-6-sulfonic acid) disodium salt; ROS, reactive oxygen species; RNS, reactive nitrogen species; TLR4, toll-like receptor 4; NF-κB, nuclear factor-κB; SOD, superoxide dismutase; MDA, malondialdehyde; GSH, glutathione; NO, nitric oxide; iNOS, inducible nitric oxide synthetase; Nrf2, nuclear factor erythroid 2-related factor 2; ARE, antioxidant response element.

In conclusion, this is the first study demonstrating that EEIH had a favorable pharmacological intervention in the prevention of LPS-induced kidney injury with potent anti-inflammatory and antioxidant activities. EEIH pretreatment dose-dependently improved renal pathological changes, inflammatory reactions, as well as oxidative/nitrosative stress. These curative effects of EEIH might be achieved by downregulating the TLR4 and NF-kB pathways and upregulating Nrf2 expression (Figure 4). These findings offer novel insights into this promising candidate for treating sepsis-associated kidney damage. However, the current study was only performed using an acute kidney injury male BALB/c model, so large-scale acute or chronic kidney injury models in other animals should be investigated. Further, LPS-induced acute kidney injury in a mouse model is a procedure with quick onset and development. Therefore, to better understand and elucidate LPS-induced AKI, continuing long-term follow-up investigations are essential for further evaluation of the clinical benefits.

## Figures and Tables

**Table 1:** Primers used for qRT-PCR.

Gene	Primer Sequence	Product Size (bp)
TNF-α	5′-CCACCACGCTCTTCTGTCTAC-3′ 5′-GAGGGTCTGGGCCATAGAA-3′	104
IL-6	5′-ACAACCACGGCCTTCCCTACTT-3′ 5′-CACGATTTCCCAGAGAACATGTG-3′	129
IL-1β	5′- GTAATGAAAGACGGCACACC -3′ 5′- CTTTGGGTATTGCTTGGGAT -3′	98
iNOS	5′-GGCAGCCTGTGAGACCTTTG-3′ 5′-GCATTGGAAGTGAAGCGTTTC-3′	72
COX-2	5′- GCAGATGACTGCCCAACTC-3′ 5′- CAGGGATGAACTCTCTCCGT-3′	103
TLR4	5′-GTTGCAGAAAATGCCAGGATG-3′ 5′-CAGGGATTCAAGCTTCCTGGT-3′	101
NF-κB p65	5′-ACGACATTGAGGTTCGGTTC-3′ 5′-ATCTTGTGATAGGGCGGTGT-3′	124
Nrf2	5′-AGCCAGCTGACCTCCTTAGA -3′ 5′-AGTGACTGACTGATGGCAGC-3′	131
GAPDH	5′-ATCCTGTAGGCCAGGTGATG-3′ 5′-TATGCCCGAGGACAATAAGG-3′	113

**Table 2:** Effects of the ethanol extract of *Illicium henryi* (EEIH) on body weight and renal index of lipopolysaccharide (LPS)-treated mice.

Groups	Dose (mg/kg)	Body Weight (g)		Renal Index (g/100 g Body Weight)
		Before Treatment	After Treatment	
NC	−	21.54 ± 0.92	24.74 ± 1.82	1.43 ± 0.21
MC	−	21.38 ± 0.94	24.18 ± 1.50	1.72 ± 0.10 ^#^
DEX	1.8	21.94 ± 1.14	24.44 ± 2.00	1.51 ± 0.11 *
EEIH	1.25	21.19 ± 0.97	25.09 ± 1.97	1.62 ± 0.12
	2.5	21.61 ± 1.03	24.81 ± 1.30	1.60 ± 0.13
	5.0	22.04 ± 0.74	25.14 ± 2.34	1.53 ± 0.13 *

The values are presented as the means ± SD (*n* = 5). Significant differences compared to the normal control group (NC) are designated as ^#^ *p* < 0.05; those compared to the model control group (MC) as * *p* < 0.05. DEX: dexamethasone (positive control).

**Table 3:** Effects of EEIH on the renal myeloperoxidase (MPO) activity and serum creatinine (Cre) and blood urea nitrogen (BUN) levels in LPS-treated mice.

Group	Dose (mg/kg)	MPO (U/g Tissue)	Cre (µmol/L)	BUN (mmol/L)
NC	−	0.81 ± 0.20	24.00 ± 1.58	8.97 ± 1.20
MC	−	9.77 ± 0.73 ^###^	55.60 ± 2.70 ^###^	29.95 ± 1.96 ^###^
DEX	1.8	1.09 ± 0.26 ***	37.00 ± 3.54 ***	16.92 ± 1.46 ***
EEIH	1.25	0.94 ± 0.38 ***	31.00 ± 1.83 ***	19.98 ± 2.11 ***
	2.5	0.84 ± 0.30 ***	29.60 ± 2.07 ***	18.50 ± 1.73 ***
	5.0	0.87 ± 0.31 ***	27.20 ± 2.39 ***	16.12 ± 1.24 ***

The values are presented as the means ± SD (*n* = 5). Significant differences compared to the normal control group (NC) are designated as ^###^ *p* < 0.001; those compared to the model control group (MC) as *** *p* < 0.001. DEX: dexamethasone (positive control).

**Table 4:** Effects of EEIH on the level of proinflammatory cytokines in renal tissue of LPS-treated mice.

Group	Dose (mg/kg)	TNF-α (pg/mg)	IL-1β (pg/mg)	IL-6 (pg/mg)
NC	−	80.62 ± 2.28	64.69 ± 3.94	105.40 ± 3.94
MC	−	140.40 ± 5.15 ^###^	135.54 ± 8.20 ^###^	168.74 ± 7.23 ^###^
DEX	1.8	98.13 ± 8.34 ***	100.53 ± 9.48 **	135.12 ± 5.99 **
EEIH	1.25	96.13 ± 4.28 ***	97.49 ± 3.97 ***	131.43 ± 9.67 **
	2.5	92.69 ± 7.54 ***	83.22 ± 6.84 ***	128.25 ± 6.70 ***
	5.0	84.74 ± 5.65 ***	77.15 ± 5.34 ***	119.16 ± 9.35 ***

The values are presented as the means ± SD (*n* = 5). Significant differences compared to the normal control group (NC) are designated as ^###^ *p* < 0.001; those compared to the model control group (MC) as ** *p* < 0.01 and *** *p* < 0.001. DEX: dexamethasone (positive control).

**Table 5:** Effects of EEIH on the mRNA expression levels of proinflammatory factors in renal tissue of LPS-treated mice.

Group	Dose (mg/kg)	TNF-α	IL-1β	IL-6	COX-2
NC	−	1.00 ± 0.32	1.00 ± 0.19	1.00 ± 0.61	1.00 ± 0.46
MC	−	31.46 ± 2.55 ^###^	13.75 ± 0.93 ^###^	260.1 ± 20.8 ^###^	26.84 ± 2.34 ^###^
DEX	1.8	18.94 ± 2.07 ***	9.24 ± 0.64 ***	166.2 ± 25.2 ***	15.54 ± 2.29 ***
EEIH	1.25	18.51 ± 2.41 ***	5.67 ± 1.11 ***	178.9 ± 22.4 ***	14.04 ± 1.52 ***
	2.5	15.17 ± 1.60 ***	5.29 ± 0.80 ***	188.3 ± 10.8 ***	13.74 ± 1.70 ***
	5.0	12.29 ± 2.10 ***	4.02 ± 0.61 ***	112.5 ± 14.0 ***	11.41 ± 1.54 ***

The values are presented as the means ± SD (*n* = 5). Significant differences compared to the normal control group (NC) are designated as ^###^ *p* < 0.001; those compared to the model control group (MC) as *** *p* < 0.001. DEX: dexamethasone (positive control).

**Table 6:** Effects of EEIH on oxidative and nitrosative stress in renal tissue of LPS-treated mice.

Group	Dose (mg/kg)	SOD (U/mg prot)	GSH (µmol/g prot)	MDA (nmol/mg prot)	NO (µmol/g prot)
NC	−	34.26 ± 1.79	10.59 ± 0.97	2.50 ± 0.28	0.24 ± 0.06
MC	−	22.32 ± 2.92 ^###^	6.57 ± 0.53 ^###^	5.43 ± 0.43 ^###^	1.01 ± 0.05 ^###^
DEX	1.8	30.82 ± 3.21 **	12.69 ± 0.78 ***	2.99 ± 0.45 ***	0.29 ± 0.06 ***
EEIH	1.25	45.07 ± 3.77 ***	14.08 ± 0.70 ***	4.47 ± 0.42 **	0.26 ± 0.03 ***
	2.5	42.78 ± 3.14 ***	15.88 ± 1.11 ***	3.51 ± 0.32 ***	0.25 ± 0.06 ***
	5.0	47.59 ± 3.79 ***	16.89 ± 0.68 ***	2.80 ± 0.25 ***	0.24 ± 0.05 ***

The values are presented as the means ± SD (*n* = 5). Significant differences compared to the normal control group (NC) are designated as ^###^ *p* < 0.001; those compared to the model control group (MC) as ** *p* < 0.01 and *** *p* < 0.001. DEX: dexamethasone (positive control).

**Table 7:** Effects of EEIH on the mRNA expression levels of inducible nitric oxide synthase (iNOS) and nuclear factor erythroid 2-related factor 2 (Nrf2) in renal tissue of LPS-treated mice.

Group	Dose (mg/kg)	iNOS	Nrf2
NC	−	1.00 ± 0.42	1.00 ± 0.21
MC	−	23.68 ± 1.39 ^###^	2.00 ± 0.24 ^###^
DEX	1.8	9.62 ± 1.72 ***	3.16 ± 0.13 ***
EEIH	1.25	8.26 ± 1.53 ***	3.26 ± 0.22 ***
	2.5	7.11 ± 1.99 ***	3.67 ± 0.23 ***
	5.0	4.38 ± 0.48 ***	4.09 ± 0.20 ***

The values are presented as the means ± SD (*n* = 5). Significant differences compared to the normal control group (NC) are designated as ^###^ *p* < 0.001; those compared to the model control group (MC) as *** *p* < 0.001. DEX: dexamethasone (positive control).

## Supplementary Materials

The following are available online at https://www.mdpi.com/2072-6643/11/6/1412/s1, Table S1: The in vitro antioxidant activity of EEIH.
