# Supplementary material for: Ethanol Extract of Illicium henryi Attenuates LPS-Induced Acute Kidney Injury in Mice via Regulating Inflammation and Oxidative Stress
Source: Nutrients. 2019 Jun 23;11(6):1412. doi: 10.3390/nu11061412 (PMC6627762; doi:10.3390/nu11061412)
Supplement: Supplementary file 1 [file nutrients-11-01412-s001.pdf]

Supplementary Materials

Table S1. The in vitro antioxidant activity of EEIH.

Plant Extract	ABTS	DPPH	FRP
Activities (mg VCE/g)	141.55 ± 7.39	22.0 ± 1.26	10.05 ± 0.07

The values are expressed as mean ± SD (n = 3). ABTS: ABTS radical scavenging activity; DPPH: DPPH radical scavenging activity; FRP: ferric reducing power; VCE: vitamin C equivalents;
